# Mechanosensitive Gating of Kv Channels

**DOI:** 10.1371/journal.pone.0118335

**Published:** 2015-02-13

**Authors:** Catherine E. Morris, Emil A. Prikryl, Béla Joós

**Affiliations:** 1 Ottawa Hospital Research Institute, Ottawa, Ontario, Canada; 2 Department of Physics, University of Ottawa, Ottawa, Ontario, Canada; Dalhousie University, CANADA

## Abstract

K-selective voltage-gated channels (Kv) are multi-conformation bilayer-embedded proteins whose mechanosensitive (MS) *P_open_*(V) implies that at least one conformational transition requires the restructuring of the channel-bilayer interface. Unlike Morris and colleagues, who attributed MS-Kv responses to a cooperative V-dependent closed-closed expansion↔compaction transition near the open state, Mackinnon and colleagues invoke expansion during a V-independent closed↔open transition. With increasing membrane tension, they suggest, the closed↔open equilibrium constant, *L*, can increase >100-fold, thereby taking steady-state *P_open_* from 0→1; “exquisite sensitivity to small…mechanical perturbations”, they state, makes a Kv “as much a mechanosensitive…as…a voltage-dependent channel”. Devised to explain successive *g*_*K*_ *(V)* curves in excised patches where tension spontaneously increased until lysis, their *L*-based model falters in part because of an overlooked *IK* feature; with recovery from slow inactivation factored in, their *g(V)* datasets are fully explained by the earlier model (a MS V-dependent closed-closed transition, invariant *L≥4*). An *L*-based MS-Kv predicts neither known Kv time courses nor the distinctive MS responses of Kv-ILT. It predicts Kv densities (hence gating charge per *V*-sensor) several-fold different from established values. If opening depended on elevated tension (*L*-based model), standard *g*_*K*_ *(V)* operation would be compromised by animal cells’ membrane flaccidity. A MS V-dependent transition is, by contrast, unproblematic on all counts. Since these issues bear directly on recent findings that mechanically-modulated Kv channels subtly tune pain-related excitability in peripheral mechanoreceptor neurons we undertook excitability modeling (evoked action potentials). Kvs with MS V-dependent closed-closed transitions produce nuanced mechanically-modulated excitability whereas an *L*-based MS-Kv yields extreme, possibly excessive (physiologically-speaking) inhibition.

## Introduction

### Overview

Kv channels which are voltage-gated channels (VGC). Their four voltage-sensing domains are biological hydrophobic cations bearing well-hydrated charge in close proximity to the embedding bilayer’s hydrophobic interior [1,2]. Via fine positional control the sensors’ motions let them regulate pore openness according to membrane voltage [3]. Perhaps not surprisingly, bilayer deformations modulate Kv activities [4,5]. Native bilayers are far-from equilibrium structures of asymmetric lipid leaflets and complex lateral arrangements. Lateral pressure profiles for simple bilayers vary strikingly depending on lipid constituents, a reminder that bilayer structure impacts the energetics of membrane proteins [6,7]. Imposed bilayer deformations that alter that structure (thickness, lipid-packing order, local curvatures and so on) can, therefore, potentially modify protein’s conformational stabilities. A bilayer’s profile can change irreversibly (chemical constituent changes, bleb-type denaturing of lipid organization [8]) or reversibly (reversibly imposed stretch, hyperbaric pressure, temperature changes, surface active molecules [7,9,10]). Membrane proteins, be they, say, rhodopsin [11] or VGCs are mechanosensitive (MS) if their conformational equilibria are modulated by such bilayer mechanical changes [4,7,12,13,14,15]; electrophysiologically speaking, an ion channel whose open probability (*P*_*open*_) is sensitive to bilayer structure [5,16] is a MS channel.

Though MS channels are generally studied using pipette aspiration to increase bilayer tension in plasma membrane patches, tension control is not straightforward [17,18] as re-emphasized by Schmidt et al [19]. Membrane/glass adhesion forces associated with gigaohm seal formation [20] combined with residual pipette pressures [21] result in “resting” patch tensions of uncertain magnitude. Moreover, insofar as seal formation disrupts cytoskeleton/bilayer adhesions and other cellular processes that maintain native bilayer structures *in situ*, it inflicts bleb-type damage (denatured bilayer structure [8,22]). But even if the bilayer denatures and acquires a non-zero resting tension, channels in patches can remain functional. If their activity or responses to stimuli become irreversibly altered [23,24,25,26], this represents a patch artifact [23] or epiphenomenon.

**Fig 1:**
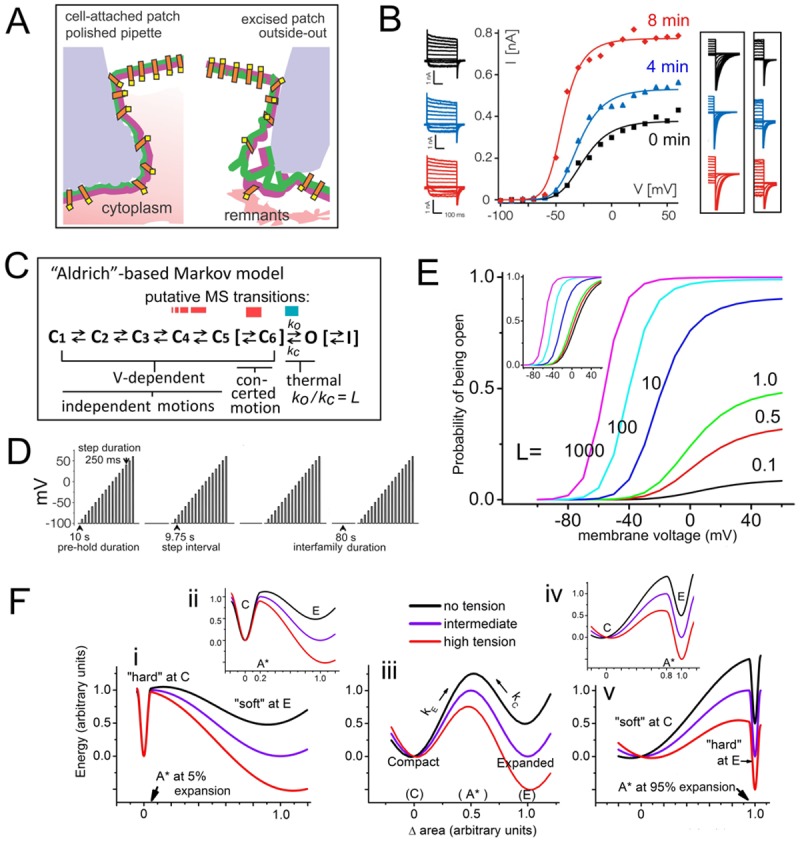
Kv channels and their mechanosensitivity. **A**. Cartoon of some differences at the gigaohm seal zones for (**left**) cell-attached versus (**right**) excised outside-out oocyte patches. **Left**, the uneven surface (pale blue) depicts pipette tip glass sputtered with molten soda glass during fire polishing [18]. **Right**, outside-out patches (whose structure would resemble that of pinched off blebs or multi-lipid artificial bilayers or [30,31]) reseal within the pipette’s unmodified borosilicate interior surface, then creep inward, developing progressively higher tension [19]. Membrane-glass interactions are extensive and stable at soft-glass sputtered tips (**Left**) and spontaneous tension build-up is less evident [17]; patches withstand multiple prolonged bouts of applied suction [32,33,34,35]. **B**. Exemplar data from Schmidt et al [19] (modified from their Fig. 1) showing “conversion” for Kv-Shaker, plus (boxed) tail currents for other Kvs. **C**. A kinetic scheme for Kv channels, extending *Scheme 1* with optional addition (square brackets) of C_6_ (“pre-open”) and I (slow inactivated) states. Putative MS transitions color-coded red for V-dependent (variants explained in the text), blue for thermal. **D**. *V(t)* clamp protocol for acquiring *g(V)* data (from “tail-I_K_”): as per [19], except that to confirm trends we simulated acquisition of 4 *g(V)*s (not 3). **E**. Effect of varying *L* on *P*_*open*_ *(V)* (values from Ref. [36]); inset, same curves normalized to *P*_*open-max*_ (similar to Fig. 2 of ref [37]). **F, i-v**. Reaction co-ordinate (drawn by eye) depicting MS transition energetics. Some area, A*, between area, C, of the smaller compacted and area, E, of the larger expanded protein conformation will be the area of the unstable “activated” or transition state [38] (for V-dependent transitions, A*-C compares to the equivalent charge moved through the electric field to put a two-state V-sensor in its transition state; see [37]). A MS channel transition could be between two distinct closed states [38,39], or, as per Schmidt et al [19] a closed and an open state. Activation barriers at the extrema, A* = C and A* = E, would imply extreme rigidities at those locations. Protein energetics depicted here at zero, intermediate and high membrane tensions (**i, iii, v**) are for A* marginally >C (5% of its expansion), or A* midway between C and E (50% of its expansion), or A* marginally <E (95% of its expansion), with two cases (**ii, iv**) of moderate barrier asymmetry (20% and 80% area expansion) as insets. In *vMS* and *LMS* models here (as in a previous MS-Kv channel model [39]) symmetry is implied (A*midway between C and E) as it is the simplest assumption in the absence of structural data for transition states (see also [38,40,41,42]).

For MS channels, patch epiphenomena involving irreversibly altered gating have been acknowledged (or dodged) with terms like “hysteresis”, “patch history” and even “exercising the patch” [27,28]. Except where putatively mechanical effects are explicitly demonstrated as reversible, it should not be assumed that the epiphenomenon represents a MS gating phenomenon [29], a caveat relevant to a patch clamp epiphenomenon reported by Schmidt et al [19], i.e., “conversion”. Conversion describes irreversible changes in macroscopic Kv channel current recorded from outside-out oocyte patches [19], the reconfigured components of which are depicted in Fig. 1A **(right)**.

Conversion is attributed to spontaneous progressive increases in bilayer tension leading to patch rupture; purportedly conversion reflect an overlooked aspect of MS gating in Kv channels[19]. The epiphenomenon is a progressive and irreversible increase in apparent *gK*_*max*_, accompanied by slope-steepening and by hyperpolarizing shifts (“left-shifts”) of *gK(V)* (Fig. 1B). Sometimes, irreversibly-increasing patch tension was augmented by pipette suction. Their native bilayer arrangements [14] degraded, the outside-out patches (Fig. 1A) underwent tension-inducing [28] patch-creep [19] (see, however, Ref. [17]). We concur that patches were under increasing tension and that this would explain part of the “conversion” epiphenomenon (specifically, left-shifted activation [39]), but the apparently novel MS aspect of “conversion” (i.e., the growth in apparent *g*_*max*_), we argue, can be explained by recovery from slow inactivation.

In their studies of MS Kv gating, Morris and colleagues [39,43,44,45] used the more robust cell-attached patch configuration (Fig. 1A) and focused principally on demonstrably reversible *I*_*K*_ time courses before, during and after applied stretch (pipettes suction) over a wide range of voltages. Though the outside-out patches of Schmidt et al [19] did not allow for relief of tension, they regard conversion as an inherently reversible MS phenomenon, modeling it as such based on g*(V)* data. Here, we use computations to consider their findings and model in comparison to those of Morris and colleagues (for several Kv Shaker constructs).

Mechanisms of MS-Kv gating have physiological implications since mammalian nociceptor neurons use Kv1 mechanosensitivity to mechanically modulate pain signals [46] and atrial rhythmicity is putatively under mechanical regulation via MS Kv7 gating [47]. With the neuronal case in mind, we checked the impact of subjecting to “stretch” a model excitable membrane comprised in part with a MS *g*_*K*_ *(V)*. We used either 1) the Schmidt et al [19] model (Kvs had a MS V-insensitive closed↔open transition) or 2) a model like that of Tabarean and Morris [39] (Kvs had a MS V-dependent concerted closed↔closed transition). The Schmidt et al MS-Kv powerfully inhibited excitability while the other model mechanically modulated excitability in a smoothly graded fashion.

Our analysis does not favor the proposition [19] that Kv mechanosensitivity can be attributed to a MS voltage-insensitive opening transition. But while a MS voltage-dependent closed-closed transition [39] is a better explanation for MS-Kv gating in Shaker type channels, it cannot be proffered as a global explanation for MS-Kv gating phenomena, let alone for MS gating in all VGCs. Other explanations are needed for the voltage-independent MS acceleration of slow inactivation [44] and for MS gating in members of the Kv3 subtype [29,45]. The putatively MS gating of a Kv7 channel [47] seems worth re-examining in light of the two classes of models addressed here. A key point emerging from the present analysis is that even though membranes cannot be “tension clamped”, hypothesis testing about putatively MS gating transitions is possible where reversible MS responses have been obtained for time courses (kinetics) and equilibria over a range of known voltages and imperfectly known tensions.

## Models for MS-Kvs

Two mechanisms have been proposed for the mechanosensitivity of Shaker type Kv channels, both assuming a transition in which the channel expands↔compacts in the plane of the bilayer, with the expanded conformation energetically favored by increased tension. In modeling these MS transitions here (as previously [39]), a simple assumption was used for the channel’s condition when in its transition (or “active”) state, A*, namely that it displaces an area halfway between the areas of the compacted (C) and expanded (E) states, as depicted in Fig 1 Fiii. As discussed previously [36], qualitatively different behaviors result if, instead of being roughly balanced, deformation into A* is heavily biased towards either the compacted or expanded state (Fig 1F i,v). Via MS rates, barrier characteristics would be apparent in *I*_*K*_ time courses, as will be pointed out as appropriate.

An earlier model (Fig. 1C, red), *vMS*, posits expansion associated *voltage*-dependent closed-closed transition(s) near the open state [39]. The new model (Fig. 1C, blue), *LMS*, posits expansion during the voltage-independent closed-open transition [19]. For a MS VGC, the issue of whether or not a MS transition involves the conductive state is pivotal, and with a strictly MS opening (the basic *LMS* model) *P*_*open*_ would only reach its operational maximum at elevated membrane tension.

Unlike the *LMS* model, the *vMS* models explored here are not confounded by the oft confirmed [48,49,50,51,52,53] fixed *P*_*open-max* =_ ~0.8 feature of Shaker Kv channels, thought to reflect flickery transitions off the voltage-activation path [49,51]. Thus, following Smith-Maxwell et al [36], the 2002 “TabMor” version of *vMS* [39] was able to ignore *L* (by making it large, i.e., 80) and the flickery open state being irrelevant in that context was not included. For the *L-*based MS-Kv model, however, large MS variations in *L* are precisely what underlie the (putative) MS increase in apparent *P*_*open-max*_ and by their reckoning (given the fits in [19]), *in situ* rest tension *L* values should be ~0.5 or less. As a compromise resolution where direct comparisons were made between *vMS* and *LMS* (see also Section Excitability and a MS-Kv: *vMS* versus *LMS*), we used *L* = 4. This would correspond to a “pre-stressed” rest-tension *L* value for *LMS* and it corresponds to *P*_*open-max*_ = 0.8. At *L* = 80 [36] the *LMS* rest-tension apparent *P*_*open-max*_ would approach unity, obliterating the central feature of *LMS* whereas the smaller fixed value of L in *vMS* does no violence to its central MS features (MS *gV* left-shift, no MS change in *g*_*max*_).

Markov type models [37] were used to predict *gK(V*,*t)* without and with simulated tension as prescribed by *LMS* or *vMS* models. Results are principally but not exclusively given as conductance time courses plus equilibrium *g(V)* and *Q(V)* curves. Use of *vMS* and *LMS* in connection with excitability modeling will be described further in the appropriate context.

## Results

### The *g(V)* data sets behind *LMS*

**Fig 2:**
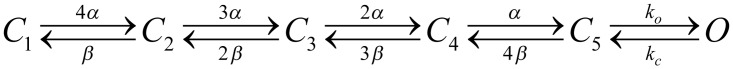
Scheme 1.

Schmidt et al [19] generated their *LMS* Kv model to account for sequentially acquired *g(V)* families (see Fig. 1B) obtained immediately upon forming outside-out oocyte patches (Fig. 1A, **right**). With patch tension increasing spontaneously for several minutes prior to rupture, their *g(V)* curves (4 min intervals; see *V(t)* protocol, Fig. 1D) grew in magnitude ~2–3 fold, with hyperpolarizing shifts and steepening slopes. In some cases, pipette suction applied between successive *g(V)s* augmented the “creep”-induced tension increases. Their *LMS* model assumes four *V*-dependent closed-closed transitions followed by a voltage-independent (“thermal”) opening, *C*_*5*_ *→O*, shown in Fig. 2 as *Scheme 1*.

The kinetic constants α and β can be expressed (using values at *V* = 0 mV) as:

$$\alpha =\alpha_{0}e^{z_{\alpha }FV/RT}=e^{z_{\alpha }F(V-V_{0.5})/RT};\beta \text{=}\beta_{0}e^{-z_{\beta }FV/RT}=e^{z_{\beta }F(V-V_{0.5})/RT},$$ [1]

where *V*_*0*.*5*_ is the midpoint membrane voltage for activation of individual voltage sensors and *z*_*α*_ and *z*_*β*_ are effective charges for voltage sensor activation and deactivation respectively (*F*, *R* and *T* have their usual meanings). The constants used in *Scheme 1*are listed in Table 1. In *LMS*, the “pore opening” last transition, *C*_*5*_ *↔O*, is “thermal” (insensitive to voltage) but is sensitive to bilayer stretch. Assuming their *g(V)* plots represented equilibria for sequentially higher (albeit unknown) membrane tensions, Schmidt et al (2012) [19] performed global fits to the equilibrium formulation for *Scheme 1*:

$$P_{open}=\frac{{\left[\frac{K}{1+K}\right]}^{4}\cdot L}{1+{\left[\frac{K}{1+K}\right]}^{4}\cdot L}.$$ [2]

**Table 1:** Kv model for Schemes 1 and 2(*) (as presented in Fig. 4 of Ref. [36] and Figure 11 of Ref. [39]).

α_0_	1120s^-1^	z_α_	0.25e
β_0_	373s^-1^	z_β_	1.0e
k_o_	8000s^-1^	k_c_	100s^-1^
*inac	0.05s^-1^	*recov	0.005s^-1^

* refers to parameters associated with Scheme 2.

The kinetic constants are embedded in ratios, *K* and *L*. *K* characterizes the *V*-dependent activation/deactivation transitions ($K=\frac{\alpha }{\beta }=e^{zF(V-V_{0.5})/RT}$where *z = z*_*α*_ *+z*_*β*_) and *L* the voltage-independent pore opening/closing transition (*L = k*_*o*_ */k*_*c*_). In “*L*-based” Kv mechanosensitivity, the channel would displace more bilayer in its *O* than in its *C*_*5*_ state, so *k*_*o*_ and *k*_*c*_ show inverse MS responses. To globally fit a patch’s *g(V)s* to Eq. 2, they held *z* and *V*_*0*.*5*_ constant, and they varied the putatively MS equilibrium constant, *L*. Note from Eq. 2 that the maximal value of *P*_*open*_ (= *L/*(*1+L*)) increases with *L*. Given the *g(V)* characteristics of their (*LMS*) model, Schmidt et al (2012) [19] conclude that “the Kv channel is as much a mechanosensitive channel as it is a voltage-dependent channel.”

When a voltage-independent entry-to/exit-from the K^+^-conducting state was first considered [48], Zagotta et al [37] showed (their Fig. 2) how such a transition impacts the V-dependence of *gK*, plotting normalized *P*_*open*_ *(V)* for *L* from 0.1 up to 1000 (see inset, Fig. 1E). A changeable *g*_*max*_ (i.e., a ∆ apparent *g*_*max*_) would be physiologically critical, so we replotted this here without normalization (Fig. 1E). The resemblance to Schmidt et al (2012)’s Kv datasets [19] (fitted *L*-values ranged from ~0.5 to >150) is self-evident (e.g., their Fig. 1L, 0.56⇾165; their Kv-Shaker data, reproduced in our Fig. 1B, 1.8⇾62). Although as explained above variability in *P*_*open-max*_ is at odds with other measurements from excised oocyte patches (noise and unitary currents, e.g.[54]) we note that for *LMS*, *P*_*open-max*_ = 0.8 corresponds to *L*≈4.

### Recovery from slow inactivation

**Fig 3:**
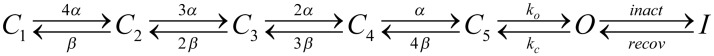
Scheme 2.

**Fig 4:**
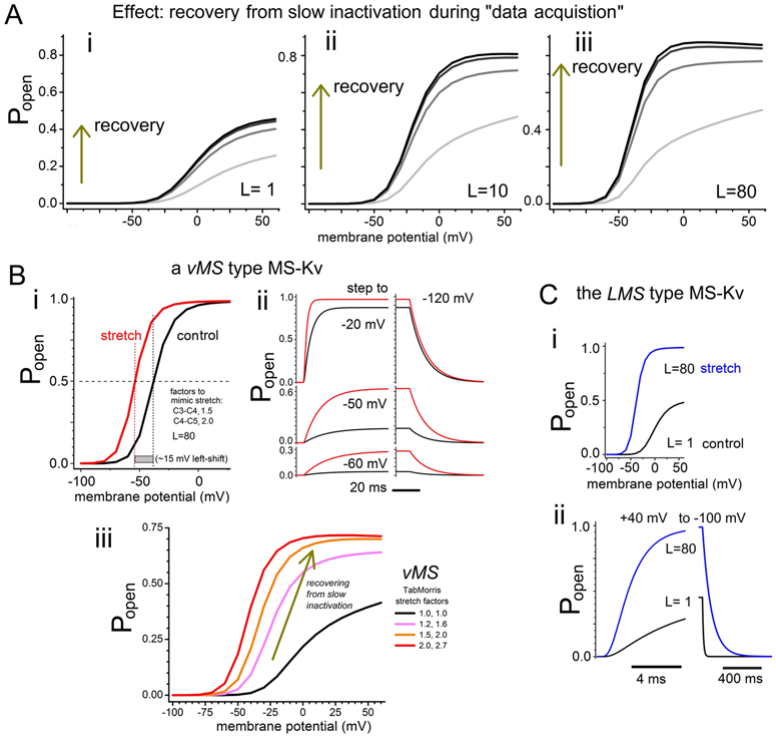
Mimicking excised patch datasets by adding slow inactivation and progressive *vMS*-type *g(V)* left-shifts to *Scheme 1*. **A, B**, and **C** are explained in the text and by figure labels. For **A** and **Biii**, the systems are initiated (i.e., t = 0, Fig. 1D protocol) from the slow inactivated state. Note (**Bi** versus **C**) that the *vMS* and *LMS* models make qualitatively different predictions for the effect of “membrane stretch” on tail time courses (i.e., closing kinetics).

Unlike previous studies of Kv mechanosensitivity from cell-attached patches (Fig. 1A) Schmidt et al [19] mostly used outside-out patches, with *V*_*m*_ = 0 mV during excision/resealing in high [K^+^] saline. A sealed patch was immediately clamped from 0 mV to *V*_*m*_ = -100 mV and data acquisition (series of *g(V)*s) started. With *V*_*m*_ = 0 mV until seal reformation, Kv channels would be slow inactivated [55]. Data acquisition occurred, therefore, in out-of-equilibrium systems. To mimic these “start” conditions, we ran the Schmidt et al [19] protocol (Fig. 1D) for *Scheme 2* (see Fig. 3), which includes the slow inactivated state, *I*:

Constants for *Scheme 2* are listed in Table 1. Fig. 4A plots outcomes for *L* = 1, 10, 80, covering much of the *L* range inferred by the Schmidt et al [19] fits. The four *g(V)*-runs we simulated show the system approaching equilibrium (recovery-from-inactivation). Schmidt *et al*, assuming tension-dependent *L* and with no mention of slow inactivation, use the term “post-conversion” for the last of their 3 *g(V)*s to signify gating under near-lytic (albeit unmeasured) patch tension.

In Fig. 4A, three *fixed L* values (1, 10, 80) are simulated; in each case, *g(V)* curves grow in amplitude with time, with upwardly inclined “plateaus” on the first *g(V)* plots and progressively steeper slope regions. Thus, recovery (more available channels) of this initially far-from-equilibrium system resembles the Schmidt et al [19] datasets, suggesting that recovery from slow inactivation (and not increasing values of *L*) underlies the ∆ apparent *g*_*max*_ in their data sets. Granted, a (tension-induced) *L* increase predicts the experimentally observed progressive *g(V)* left-shifts (note *V*-axis positions of *g(V)-*sets for *L* = 1,10,80), but so too does *vMS*. We therefore next look at a *vMS* version of *Scheme 2* operating during recovery from inactivation.

### Kv mechanosensitivity in a voltage-dependent transition

Stretch-modulation of Kv-Shaker and its short-S3-S4-linker mutant (“5aa”) in cell-attached oocyte patches was previously described with a *vMS* version (“TabMor”) of *Scheme 2* [39]. Mechanosensitivity was attributed to a closed-closed V-dependent conformation change near the open state. Tabarean and Morris [39] approximated a MS concerted pre-opening by reciprocally “stretch modulating” the forward and backward rates of the penultimate and ultimate *V*-dependent transitions of *Scheme 2* (as in the example of Fig. 4Bi MS modulation was depicted as stronger in the second transition). For a given (unspecified) tension, rates at *C*_*3*_ ↔ *C*_*4*_ and at *C*_*4*_ ↔ *C*_*5*_ change by “stretch factors” *ms*_*3↔4*_ and *ms*_*4↔5*_ respectively. Since Tabarean and Morris [39] simulated selected *g(V*,*t)* without plotting *P*_*open*_ *(V)* for control and “stretch” conditions, we rectify that here (Fig. 4Bi), calculating *g(V)* (10 mV intervals) for the parameters as labeled. Unlike *LMS*, *g*_*max*_ is unaffected as *g(V)* left-shifts with “stretch” (in those simulations, *L* = 80 following [36] but for any fixed *L g*_*max*_ is invariant). Fig. 4Bii shows that *vMS*-simulated stretch weakly modulates the rate of conductance onset but, importantly, not the offset rate (i.e., the tails).

For Fig. 4Biii, the Schmidt et al [19] *V(t)* protocol (Fig. 1D) was used with *Scheme 2* and the *vMS* parameters of Fig. 4Bi except that *L* = 4. Henceforth for *vMS*, L = 4 *= k*_*o*_/*k*_*c*_ (= 400/100). Importantly, all channels were initially in state *I*. Then, to mimic membrane tension progressively increasing above its rest (control) level, stretch factors (*ms*_*3↔4*_ and *ms*_*4↔5*_) were increased for successive *g(V)s*. The “TabMor stretch” version of *vMS* used here are as labeled; in terms of *Scheme 2* kinetic parameters, this produces a small increase in the ratio 2α/3β and a slightly larger one for α/4β. Notice how closely the resulting *g(V)* set mimics the exemplar datasets of Schmidt et al [19] (e.g. Fig. 1B, here).

Thus, using 1) a fixed *L* magnitude consistent with established values of *P*_*open-max*_ (e.g., [48,49]), and 2) realistic magnitudes for a *vMS-*type left-shift (i.e., consistent with *I(V*,*t)* data before/during/after stretch [39]), and 3) known rates of Kv Shaker slow inactivation [55], the outcome is a *g(V)* set (Fig. 4Biii) with all the features of the Schmidt et al [19] *g(V)* datasets.

### *LMS* predictions for tail currents under stretch

**Fig 5:**
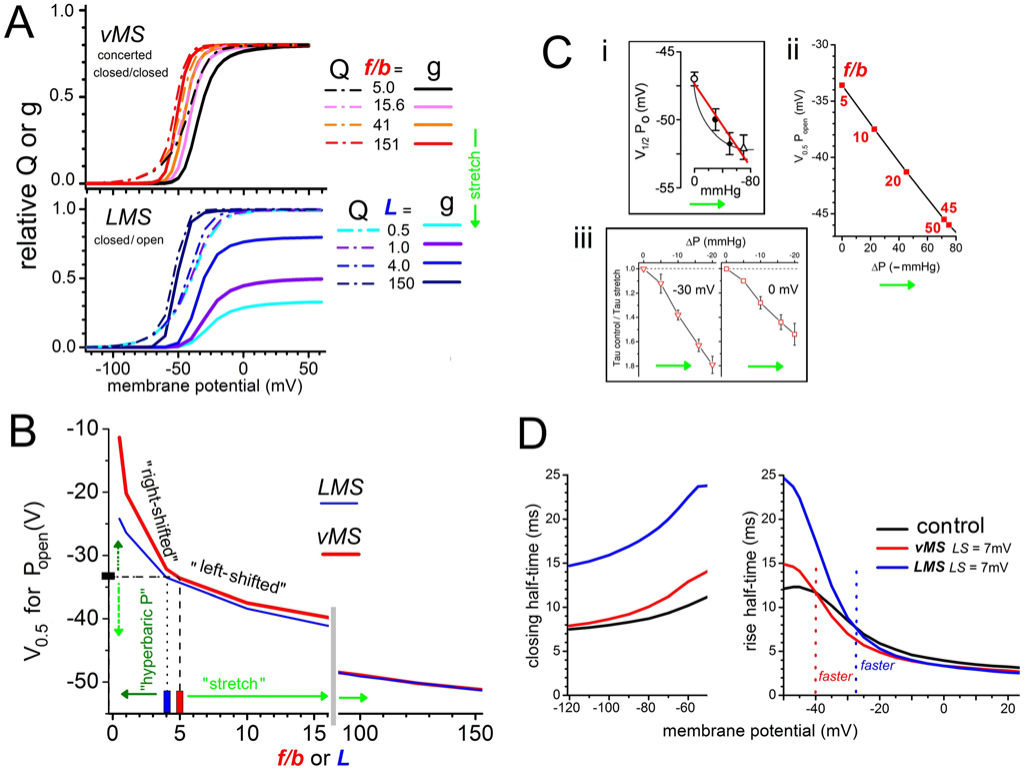
Comparing *vMS* and *LMS* using *Scheme 3* (concerted pre-opening). **A**. *g(V)*s and coinciding *Q(V)*s (normalized to *g(V)*s of *vMS*, where *P*_*open-max*_ = 0.8 because *L* = 4) at the listed equilibrium constant values. For the *vMS* case, rest tension (no applied stress) is (*f/b)*_*0*_ = 5 and for *LMS*, the rest tension *L* value would be near 1 as discussed in the text. **B**. *g(V)* midpoint (*V*_*0*.*5*_) as a function of the MS equilibrium constants for each model. **Ci**. *g(V)* left-shift data redrawn from Hao et al (2013)[46]. The red line overlay is our “by-eye” fit. **ii**, *Scheme 3 vMS-* Kv *g(V)* left-shift calculated as a function of ∆P; *P*_*open*_ (*vMS*-Kv) was modeled for a hemispherical-section patch whose increasing radius of curvature for 0 to 80 mmHg suction caused membrane-area to increase by 1.4% (the patch model was a perfect hemisphere for a 4% area-increase which is a bilayer’s typical lytic limit; other parameter values were K_A_ = 250 mN/m for bilayer stiffness, conformation change ∆area = 3 nm^2;^ patch radius = 0.5 μm). *LMS* left-shift behavior (not shown) is similarly quasi-linear. **iii**. Replotted data from Fig. 5 of Ref. [56] for MS changes in Nav1.5 fast inactivation kinetics (tight kinetic coupling makes this inactivation process a good proxy for V-dependent Nav1.5 activation; and as for Kv1, the *g(V)* for Nav1.5 left-shifts reversibly with patch stretch). **D**. Half times for Kv conductance tails and onset, for a control condition ((*f/b)*_*0*_ = 5, *L* = 4) of *Scheme 3* and after parameter changes yielding 7 mV *g(V)* left-shifts according to *vMS* and *LMS* models. Note, with *LMS*, the pronounced slowing of tail decay. Note, also, that for onset, in the *vMS* case conductance rise would be accelerated by stretch at-40 mV and beyond, but in the *LMS* case it would rise more slowly with stretch till about-27 mV.

The *LMS* model postulates a ∆-area at *C*_*5*_ *↔O*. Recall that the models used here assume an activated (transition) state, A*, symmetrically located between the compacted and expanded states, which for *LMS* would be *C*_*5*_ and *O*. The unlikely situation of A* being at or almost at the expanded state (Fig. 1Fv) will be addressed below, but ignoring that for the moment, *LMS* predicts that “tail current” (measured at hyperpolarized voltages to track closing, *O→C*_*5*_) should slow dramatically at elevated membrane tensions, as illustrated for *L* = 1 = 100/100 going to *L* = 80 = 894.4/11.20 in a *Scheme 1* simulation (Fig. 4C). Schmidt et al [19] do not address this prediction, but Kv Shaker tail currents, which have been monitored before/during/after stretch in cell-attached patches [45], show no consistent change with increased tension. Schmidt *et al’s* exemplar datasets include tail currents; inspection reveals no consistent changes as tension grows. These experimental findings agree with the *vMS* models here (e.g., Fig. 4Bi); they predict tail currents at hyperpolarized potentials almost unaffected by stretch, as in Fig. 4Bii (see also Fig. 5D).

Stretch, we note, accelerates tail current for another VGC, HCN2 (Lin et al [57]; see their Fig. 6). Though HCN channels resemble Kv channels, they close in response to depolarization and while details are sketchy, HCN tail currents should reflect V-dependent sensor motions [58]. Stretch-*accelerated* closing of HCN2 is inexplicable by a *LMS* ∆-area model whose underlying mechanism involves compaction to the closed state and expansion to the open state.

We now return to the conjecture raised earlier that Kv tail current insensitivity to stretch is explicable via *LMS* if A* = E (or say, at ~95% in its area expansion) (see Fig. 1Fv) which would make current onset (regardless of voltage) accelerate strongly with stretch and leave tail currents unaffected. For the Schmidt et al exemplars (their Fig. 1), inferred *L* values increased ~60-fold (*L* = ~1.5 for controls going to *L* = ~90 for *g(V)*) upon “conversion”. In the notation of Fig. 1F (*L = K*_*E*_ */K*_*C*_), *K*_*C*_ would be invariant with tension so current rise would be ~60-fold faster than control at the higher tension. The traces reveal no such changes and none are mentioned. By contrast, small (reversible) MS changes are seen for Shaker-type Kv rise times [39,43,44,45] along the lines summarized in Fig. 5D (red), a *vMS* depiction of what (experimentally) would represent application of near-lytic stretch (explained further in the next section; as in all modeling here, the rates for that *vMS*’s MS transition—the equivalent of Fig. 1F’s generic *K*_*E*_ and *K*_*C*_—operate over an area-symmetric energy barrier). Thus, while an *a priori* rejection of *LMS* based on the insensitivity of tail currents to stretch is too facile, MS rise time changes for Shaker channels are entirely inconsistent with such an explanation, whereas both rising current and tail current time courses patterns are fully consistent with the proposed *vMS* model.

### For both *vMS* and *LMS* models *Q(V)* limits MS left-shifts of *g(V)*

**Fig 6:**
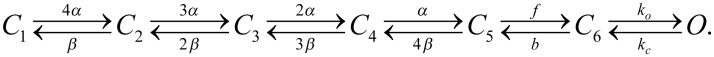
Scheme 3.

Previously [39] (the “TabMor” *vMS*) we approximated a MS concerted V-dependent pre-opening but with that transition now better understood, we can use *Scheme 3* (see Fig. 6), which includes an explicit concerted V-dependent *C*_*5*_ *↔C*_*6*_ before the open state:

Table 2 lists the constants for this 7-state scheme. The *C*_*5*_ *↔C*_*6*_ concerted voltage sensor motion (involving ~13% of total gating charge) is thought to exert a laterally-acting (centripetal) force to destabilize the cytoplasmic “bundle crossing gate” (see [35,59]). By making *C*_*5*_ *↔C*_*6*_ versus *C*_*6*_ *↔O* the sole MS transition, Fig. 5 compares the predictions of *Scheme 3-*based *vMS* versus *LMS* models.

**Table 2:** *Scheme* 3 (7-state) Kv model for WT and ILT.

	WT	ILT		WT	ILT
α_0_	2000s^-1^	1540s^-1^	z_α_	0.5e	0.5e
β_0_	100s^-1^	0.16s^-1^	z_β_	1.5e	2.5e
f_0_	1000s^-1^	0.072s^-1^	z_f_	0.2e	1.0e
b_0_	200s^-1^	0.162s^-1^	z_b_	0.8e	0.8e
k_o_	400s^-1^	400s^-1^	k_c_	100s^-1^	100s^-1^

For WT, model based on Ref. [15] equilibrium model for *g(V)* and *Q(V)*curves with kinetic constants and effective charges inspired from Ref. [50]; for ILT, charges and rate constants started from Ref. [35], but shifted to give *g(V)* and *Q(V)* midpoints consistent with those in the literature *V*_*0*.*5*_^*Q(V)*^ ≈ -80 mV and a *V*_*0*.*5*_^*g(V)*^ ≈ 100 mV (see Refs. [35] (Fig. 5), [15,60]).

$C_{6}⇄O$, the thermal transition, is the same for ILT and the WT, *L = k*_*o*_ */k*_*c*_ = 400/100, yielding *P*_*open-max*_ = 0.8.

Fig. 5A depicts relative *g(V)*s with the corresponding *Q(V)*s for *vMS* and *LMS* models for a range of “stretch intensities”. Increasing tension is mimicked by increasing *f/b* for *vMS* or by increasing *L* (= *k*_*o*_ */k*_*c*_) for *LMS*. For a tension-dependent *L*, the low end of the Schmidt et al [19] *L* values (from their global fits) would approach rest tension values. In *vMS*, *g*_*max*_ is unchanging as stretch left-shifts the *g(V)*, while in *LMS*, true rest tension *g*_*max*_ should be less than experimental estimates while at large *L* values (say 100) apparent *g*_*max*_ and true *g*_*max*_ converge (Fig. 1B). Over the inferred *L* range from Schmidt et al’s [19] fits (~ 0.5 to >150), apparent *g*_*max*_ increases ~3-fold. Note how, as the simulated “stretch intensity” grows, the left-shift of *g(V)* is increasingly limited by the *Q(V)* curve which always lies to the left of *g(V)*. Unlike the “TabMor” version of *vMS*, neither the *LMS* nor the *vMS* models assume any MS effect at the voltage sensors’ independent transitions. Nevertheless, as evident from the co-plotted *g* and *Q* curves, mechanosensitivity in either the charge-poor concerted pre-opening transition or the entirely charge-free opening transition would yield appreciable MS shift/steepening of *Q(V*). Conversely, experimentally-observed MS *Q(V)* changes of this ilk would not signify MS effects on independent voltage sensor motions.

Fig. 5B summarizes MS *g(V)* shifts for both models over as a function of their MS equilibrium constants. Rest tension values are indicated by boxes on the axes, with increasing stretch (bright green arrows) or hyperbaric pressure regimes (dark green arrows) associated with the labeled ranges. In both models, *g(V)*s left-shift under tension and right-shift under hyperbaric pressures but neither gives a tension-induced “saturation” of left-shift.

For *vMS*, the rest tension value of the equilibrium constant, *(f/b)*_*0*_ (*f/b* at *V* = 0 mV), is 5 (red marker, *x*-axis) (Fig. 5B, see labels). Although values >5 signify stretched membrane while <5 would signify compressed membrane (hyperbaric pressures right-shift Kv and Nav activation (Conti and colleagues [56], discussed in Ref. [39]) this *x*-axis should not be misconstrued as a “membrane tension” axis. The relationship between *f/b* (or *L*) and membrane tension is derived below.

For Kv1.1 channels in HEK cells, Hao et al [46] report MS *g(V)* left-shifts which, they thought, saturated at large patch pipette pressures. Part of their Fig. 5D is redrawn in Fig. 5Ci; our red line overlay shows that the data comfortably fit a straight line. This matters, since for both *vMS* and *LMS* models, a near-straight line, but definitely not a saturation, is predicted as a function of pipette pressure (Fig. 5Cii). Direct calibration of the models’ MS equilibrium constants against pipette pressure (the *x*-axis of the Hao et al [46] plot) or even against membrane tension, is not possible. Instead, we derived the Fig. 5Cii plot using simple arguments that relate the *g(V)* midpoint, *V*_*0*.*5*_, to applied pipette pressure and the MS equilibrium constants. We first note the order in which the relevant quantities can be related:

$$V_{0.5}↔\left(\frac{f}{b}\right)\;\text{or}\;L↔\gamma =K_{A}\varepsilon ↔\Delta P$$ [3]

The first two are related through Fig. 5B. Next, (*f/b*)_0_ (or *L*) are related to membrane tension γ, through a Boltzmann relationship. The ratio *f/b* (or *L = k*_*o*_ */k*_*c*_) gives the ratio of probabilities of occupation of states *C*_*5*_ and *C*_*6*_ (or *C*_*6*_ and *O*), hence:

$$\frac{{\left(f/b\right)}_{\gamma \neq 0}}{{\left(f/b\right)}_{\gamma =0}}=\exp\left(\frac{\gamma \Delta A}{k_{B}T}\right),\;\text{or}\;\gamma \text{=}\frac{k_{B}T}{\Delta A}\ln\left(\frac{{\left(f/b\right)}_{\gamma \neq 0}}{{\left(f/b\right)}_{\gamma =0}}\right),$$ [4]

where ΔA is the area change of the MS transition. This holds for all *V*, and if one assumes that stress affects the free energy of the channel only through the term (-*γ ΔA*), then the *V = 0* value of the two (*f/b*)_0_ can be used in the expressions. Next, γ is proportional to the lateral expansion of the membrane, ε, through *γ = K*_*A*_ *ε*, where *K*_*A*_ is the compressibility modulus for membrane expansion. And finally Laplace’s law relates ε to Δ*P*, the specific values depending on the initial curvature of the patch. When standard values for membrane patches are used for the various quantities, the predicted effect of applied pressure, Δ*P*, on *V*_*0*.*5*_ is as seen in the essentially linear relationship of Fig. 5Cii. For a different VGC, Nav1.5, Fig. 5Ciii shows a similar outcome in the MS rates of inactivation (Nav1.5 inactivation couples tightly to activation and thence to the MS-modulated *g(V)* midpoint [61]); as with the MS-Kv data, Δ*P* has an essentially linear effect.

Why the linearity? Although *g(V)* left-shift declines strongly as the MS equilibrium constant increases (i.e., (*f/b*)_0_ or *L* for *vMS* or *LMS* models respectively, as per Fig. 3B), *g(V)* left-shift grows exponentially with γ and consequently these behaviors largely neutralize each other. Thus, neither statistics nor theory support saturation of the MS *g(V)* shifts observed [46] for the neuronal MS-Kv.

The neuronal MS-Kv is, nevertheless, physiologically compelling, and the MS *g(V)* left-shift observed by Hao et al [46], i.e., ~7 mV at large pipette pressures, we take to represent a physiological extremum given that near-lytic tension (not “saturation”) is likely what sets the upper limit. This amount of shift accords with MS shift extrema reported for Kv-Shaker and Nav1.5 channels in oocyte patches [39,62].

From time courses (*g(V*,*t)*), Fig. 5D plots onset and tail halftimes for control (no stretch) and both *vMS* and *LMS* stretch (7 mV left-shifts in each case). For *vMS*, the rest tension value of the concerted closed-closed transition equilibrium constant, (*f/b*)_0_, was 5. To facilitate comparison, *L* = 4 was used for rest tension in both models. Had we modeled the 7 mV *g(V)* shift interval of the *LMS* case starting from say *L* = 0.5 or 1.0 (see Fig. 5B *y*-and-*x* axes for the relevant upper value for *L*) results would be quantitatively but not qualitatively different. Fig. 5D, then, compares halftimes for *vMS* vs *LMS* versions of *Scheme 3* before then after a 7 mV *g(V)* left-shift ((*f/b)*_*0*_ at 5 then 15.6 for vMS; *L* at 4 then 14.4 for *LMS*)). The key outcome: for *vMS*, the tail time course at a hyperpolarized test voltage (say, -120 mV) will be essentially insensitive to stretch while that for *LMS* will slow markedly with stretch. As explained above (section on tail currents), this *vMS* kinetic pattern is consistent with experimental observations, thereby casting strong doubt on the *LMS* model.

Some subtler features could also be useful for experimentalists. In both models, onset half times during depolarizing steps (right side of plot) go through a “crossover” voltage. Its value left-shifts as stretch intensity increases (not shown). Kinetically, *vMS* produces net stretch acceleration starting at more hyperpolarized *V*_*m*_ than with *LMS* (see labeled crossover points). Left-shifting *g(V)s* would ensure stretch-augmented steady-state currents in either models but, especially for steps to near the foot of the *g(V)*, stretch-induced time course changes could be baffling if one was unaware of the crossovers. For excitable cells, *vMS* would be a simpler mechanical tuner than *LMS*. *LMS* would be powerful yet ungainly, with stretch substantially increasing absolute *gK* (Fig. 5Aii) albeit with slower kinetics over much of the physiological voltage range (compare the crossover points). We return briefly to the relative mechano-electrical “competencies” of the two models in the last section.

### ILT mechanosensitivity: consistent with a *vMS* model, inconsistent with the *LMS* model

**Fig 7:**
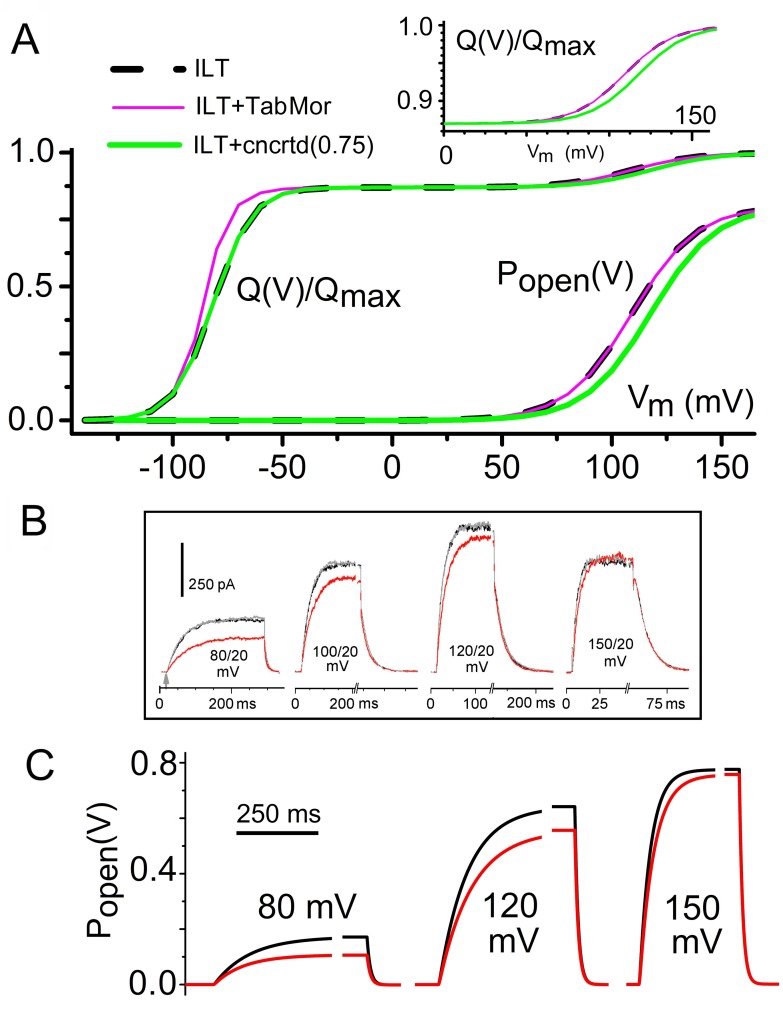
A 7-state *vMS* model of Shaker ILT: *Q(V)*, *g(V)*, and time courses. The four independent V-dependent transitions account for 87% [50] of charge movement and the concerted V-dependent closed-closed transition accounts for the remaining 13% [63],[59],[15]. Kinetic constants are Table 2. **A**. For a *Scheme 3* Kv-ILT model, the *Q(V)s* for rest tension (dashed black line) and with applied tension acting on the concerted transition (green) (*Q(V)* from 0 to 150 mV is expanded above). To illustrate that any “stretch effects” on V-dependent transitions prior to *C*_*5*_ *↔C*_*6*_ would be uncoupled from the ILT-*g(V)*, an ILT simulation (pink line) using the same “TabMor” parameters as previously (Fig. 4B) was included (Note that *P*_*open*_ *(V) = g(V)/g*_*max*_). Comparison of **B** (experimental *I*_*K*_ *(V*,*t)* data for ILT, where black, red and gray traces are before, during and after patch stretch [45]) and **C** (time course simulations, with black and red signifying rest and elevated tension, respectively) shows that a *g(V)* right-shift due to MS rate changes in the concerted transition (i.e., green in **A**) yields appropriate *g(V*,*t)* behavior.

The Kv-Shaker-ILT mutant isolates the independent voltage-driven motions of the four Kv voltage sensor domains from the ensuing concerted voltage-sensitive pre-opening [37,63,60]. In wild type (WT) Kv-Shaker, pre-opening (see *Scheme 3*) couples tightly to the last of the four independent motions. In ILT, independent charge motion is facilitated while the concerted motion is severely hampered, so the *Q(V)* component associated with independent charge movements is left-shifted even as *g(V)* and the small concerted motion *Q(V)* component right-shift. Dashed black lines in Fig. 7A depict normalized *Q*(V) and the *P*_*open*_ *(V)* for ILT; the corresponding curves for WT (not shown) both lie near-50 mV (*Q(V)* near-50mV, and *G(V)* about-25 to-40 mV) [15,35,60,63]; parameters are in Table 2. To open ILT requires extreme depolarization, perhaps because [60] altered interactions between the mutated S4 segments and the lipid bilayer, plus abnormally strong protein-protein interactions stabilize the closed bundle crossing (“activation gate”). Since open ILT and open WT channels have identical unitary conductances, opening in ILT, *C*_*6*_ *⇾O*, is presumably not to some novel conformation. Accordingly, for both ILT and WT, a *LMS* model predicts a stretch-induced increase in apparent *g*_*max*_, a left-shifted *g(V)* and slowed current offset (i.e., tail currents). Patch recordings of ILT channels show that none of these *LMS* expectations are met: with stretch, the ILT *g(V)* right-shifts, current onset slows, and tail currents are unaffected [59] (see sample currents in Fig. 7B).

Clearly, ILT is not a Kv-*LMS*. But can a *vMS* model predict ILT’s strange MS behavior? As a first point, any *vMS* model of ILT using *Scheme 3* (which terminates in a thermal closed-open transition) predicts its stretch-insensitive tail currents. However, the *vMS* explanation that worked nicely for WT does not predict ILT’s stretch-slowed current onset nor the attendant stretch-induced *g(V)* right-shift. We modeled ILT as shown in Fig. 7A then tested *vMS* variants. Not surprisingly, a *Q(V)* left-shift (done like TabMor on WT [39]) has no impact on the ILT *P*_*open*_ *(V)* curve. A *vMS* version of *Scheme 3* that does yield a ILT-like MS-*g(V*,*t)* response pattern is as follows: a stretch-induced decrease by a factor 0.75 in the forward rate of the concerted voltage-dependent transition *C*_*5*_ *↔C*_*6*_ and a corresponding increase by the same factor in the backward rate (result plotted in Fig. 7C). The corresponding *P*_*open*_ *(V)* change or *g(V)* right-shift (green) is shown in Fig. 7A. The ILT *I(V*,*t)* data [45] in Fig. 7B illustrate that MS rate changes in this range mimic large but experimentally available membrane tension increases. In both experimental and *vMS*-simulated traces, onset decelerates under stretch but tails (closing) are insensitive to stretch.

Thus, membrane stretch/deformation further slows an already difficult V-dependent closed-closed transition in ILT, a channel in which the rate-limiting V-dependent transition has been pinpointed as the next-to-last in a multi-step transition [60]. If, in both ILT and WT, a V-dependent component of a step-wise concerted transition involves an in-plane expansion closed-closed expansion (*vMS*), how might one envisage a subsequent stretch-insensitive (thermal) transition in and out of the open state? Two possibilities suggested by experimental findings are suggested in the discussion.

### Excitability and a MS-Kv: *vMS* versus *LMS*

**Fig 8:**
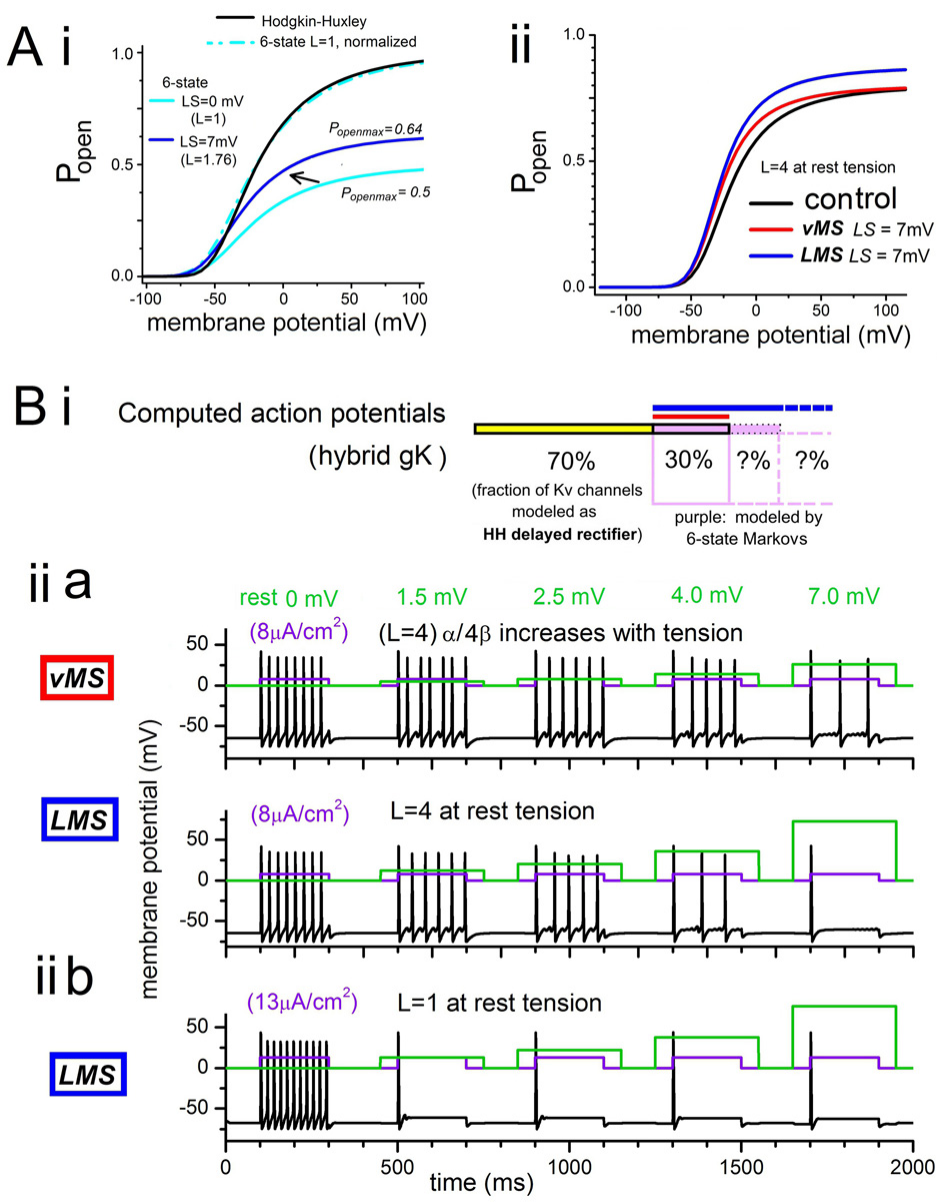
Effects of stretch-modulated MS Kv (3 versions) on electrically stimulated APs. In these excitability models, 70% of *gK*_*max*_ is a Hodgkin-Huxley (HH) type *gK* and the remaining 30% is a *Scheme 1* (6-state) Markov adjusted to approximate the HH-type *gK* with respect to *V*_*0*.*5*_ and steepness (see Table 3 for the parameter values). **Ai**. Normalized *P*_*open*_ *(V)*s for HH and 6-state systems with *L* = 1.0 (rest), plus non-normalized plots for *L* = 1.0 and *L* = 1.76 (i.e., 7 mV of *LMS* left shift). **Aii**. For the 6-state Markov using *L* = 4 as rest value, *P*_*open*_ *(V)* for that rest value and for a 7 mV left-shift (*LMS*: *L* changes from “pre-stressed rest” of 4.0 to 6.9; *vMS*: the ratio of *C*_*4*_ *↔C*_*5*_ kinetic constants *α/4β* changes by a factor of 1.76). **Bi**. Diagram of contributions to *gK*_*max*_ (two sub-populations of Kv channels as labeled). **Biia**. Responses of the *vMS* and *LMS* excitability systems to an 8 μA/cm^2^ stimulus, with rest tension (*LS* = 0) corresponding to *L = k*_*o*_ */k*_*c*_ = 400/100 = 4.0. MS left-shifts (*LS)* are modeled by accelerating the forward rate by the same factor by which the backward rate is reduced. The *LS* values (1.5, 2.5, 4.0, 7.0 mV) are obtained as follows: for *vMS*, *α/4β* increases by factors 1.05, 1.079, 1.14, 1.26 respectively and for *LMS*, with *L* = 4.5, 4.8, 5.4, 6.9 respectively (green lines represent % increases of *α/4β* or *L*). **Biib**. As in **Biia**, for *LMS* but with *L* = *k*_*o*_ */k*_*c*_ = 100/100 = 1.0 at *LS = 0* and *L* = 1.13, 1.22, 1.38, 1.76 for the other values of *LS* = 1.5, 2.5, 4.0, 7.0 mV respectively. *I*_*stimulus*_ for this case, 13 μA/cm^2^.

For excitable membranes that experience tension fluctuations, MS-Kvs could be physiological modulators. MS-Kv7 channels, for example, might signal strong atrial myocyte distension [33]. Especially pertinent here neuronal MS-Kv1.1 alluded to already; in pain sensing mechanoreceptor neurons, it can mechanically tune the characteristic mechanosensory firing patterns [46]. The recombinant channels exhibit MS-*g(V)* left-shifts of up to 7 mV, but since kinetic specifics in the neuronal *gK(V*,*t)* have yet to be studied detailed neuronal modeling would thus be premature. Basic comparisons of *vMS*-Kv versus *LMS-*Kv performance in a simple excitability setting are, however, warranted. To do so, we substituted a fraction (30%) of a Hodgkin-Huxley (HH) [64] system’s *g*_*K*_ with a Markov *g*_*K*_ that distinguishes the two putative MS-Kv types (see Appendix). Fig. 8Ai,ii plots the various *g*_*K*_ *(V)*s.

For this purpose, *MS-g(V)s* were described using *Scheme 1* (“6-state”) (Fig. 8Bi) with stretch modulation affecting only *C*_*4*_ *↔C*_*5*_ (*α/4β*) for *vMS*, and only *C*_*5*_ *↔O* (*L* = *k*_*o*_ */k*_*c*_) for *LMS*. Use of *L* = 4 at rest tension allowed for direct inter-model comparison (Fig. 8Biia). In another simulation, use of a lower value (*L* = 1 (Fig. 8Biib)) approximated more closely what Schmidt et al [19] would see as a near-physiological rest tension *L* value. We reverted to *Scheme 1* in these excitability tests for electrophysiological simplicity because *Scheme 3* differs too much from HH; each of the two “rest tension (control)” *MS*-*gK(V)* used were adjusted to closely match the HH-*gK(V)*. Parameters are listed in Table 3. Mechanical stimuli would deform only part of an excitable cell (e.g., in the Hao et al [46] DRG neuron clamp experiments); for this reason the *vMS-* or *LMS-*modulated *gK* were made to account for only part of total rest-tension *gK*_*max*_ (30% was chosen arbitrarily) (Fig. 8Bi). Fig. 8Ai shows the HH *g(V)* and an *L* = 1 version of *LMS-gK(V)* normalized to each other (black and dashed turquoise lines). As depicted in Fig. 8Bi, for the *L*-based MS-Kv (i.e., *LMS*), the excitability model deals with *gK*_*max*_ as an apparent value (see Eq. 2 and the Appendix); under stretch conditions, it exceeds its rest-tension value (as for the 7-state *LMS* Markov of Fig. 5A, intense enough stretch, apparent *gK*_*max*_ could triple if rest tension *L* ~0.5, albeit not for a 7 mV *g(V)* shift). Fig. 8Aii shows *g(V)s* for “rest tension” (control, *L* = 4) then after a 7 mV left-shift (“LS”) produced by increased *L* or increased *α/4β*.

**Table 3:** Parameters for two sub-populations of Kv (one MS, one not) used to study the effect of a MS-Kv on HH type excitability.

	HH model(s^-1^)(*V* in mV)	*Scheme 1* with L = 4(s^-1^)(*V* in mV)	*Scheme 1* with L = 1(s^-1^)(*V* in mV)
α_n_	$10\frac{\left(V+55\right)}{1-\exp\left(-\frac{V+55}{10.0}\right)}$	$\frac{10}{1.55}\frac{\left(V+55\right)}{1-\exp\left(-\frac{V+55}{12.0}\right)}$	$\frac{10}{1.55}\frac{\left(V+18+55\right)}{1-\exp\left(-\frac{V+18+55}{12.0}\right)}$
β_n_	$125\exp\left(-\frac{V+65}{80}\right)$	$1.55\times 125\exp\left(-\frac{V+65}{85}\right)$	$1.55\times 125\exp\left(-\frac{V+18+65}{85}\right)$
k_o_	not applicable	400	100
k_c_	not applicable	100	100

Fig. 8 Biia *(vMS vs LMS)* and 8Biib *(LMS*, *L* = 1) show action potentials (APs) elicited by fixed intensity depolarizing currents (excitation threshold is higher for 8Biib, hence the larger *I*_*stim*_). In Fig. 8Biia the excitability model responds to a marginally supra-threshold *I*_*stim*_ (on/off as per purple lines) first without stretch at rest tension (i.e., “*LS*” = 0 mV) then with stretch described by *vMS* or by *tM S* (stretch “on/off “as per green lines) to give 1.5, 2.5, 4 or 7 mV of left-shift (*LS*). The corresponding *α/4β* and *k*_*o*_ */k*_*c*_ *= L* changes are listed in the legend. For rest tension proportion of MS-*gK* greater (or less) than the 30% used here, MS impacts on excitability would be more (or less) powerful than shown. Since it would be inconsistent with the Schmidt et al [19] analysis for the *LMS* model to have *L* = 4 at rest tension (4 would, by their reckoning, correspond to a high membrane tension) this could be regarded as a “pre-stressed” condition. Direct comparison between models requires this since in *vMS*, *L* = 4 at all stresses. With that proviso, Fig. 8Biia shows that for both models, increasing “stretch intensity” causes a graded inhibition of AP firing frequency. With *L* = 4 at rest tension in both models, outcomes for *LMS* and *vMS* are not substantially different though double-faceted *MS* (left-shift plus ↑apparent *g*_*max*_) inhibits the electrically stimulated APs slightly more than does *vMS*.

Next, to compare *LMS* and *vMS* when neither has an appreciable pre-stress, we look to the lower rest tension *L* simulation. For true rest tension, *L* should, by the arguments of Schmidt et al, be lower than their smallest *L* values since their patches are assumed to have elevated rest tensions. Nevertheless, we chose *L* = 1, i.e., near the low end of their fitted range. As Fig. 8Biib shows, this puts an outright brake on excitability at the smallest tested “stretch” intensity. Any nuance disappears; even for the lowest “stretch intensity” tested (equivalent to a 1.5 mV left-shift), the stretch-induced apparent g_*max*_ increase of this *LMS* extinguishes excitability. By contrast, in the *vMS* scenario (top line of Fig. 8Biia), over the 1.5–7 mV MS-left-shift range, the AP patterns convey information about mechanical stimulus intensity and duration. We again point out however, that “braking” and modulation efficacy would depend dramatically with the proportion of total *gK* experiencing stretch. Additionally, MS braking requirements increase substantially once a MS-cation channel is present. Once additional native cell MS-Kv kinetic data become available, the approach used here could incorporate those features along with a MS-*g*_*cation*_ and the cell’s full complement of channels.

## Discussion

### Overview

In 2002 it was proposed that Kv mechanosensitivity [39] is explained by a cooperative voltage-dependent closed-closed transition near the open state and structure function studies showed that the Kv pore is more stable closed than open so “to open the pore the voltage sensors must exert positive work by applying an outward lateral force near the inner helix bundle” [66]. But interestingly, Mackinnon and colleagues recently argued that the final voltage independent transition in/out of the conducting state, not a preceding voltage-dependent transition, accounts for Kv mechanosensitivity [19]. Here, we addressed the known mechano-electrophysiological behaviors of Kv channels in light of these two mechanistic models. Because “tail current” decay would almost certainly be stretch-sensitive in one case but not in the other, the demonstrated stretch-insensitivity of tail currents favors the latter model. We nevertheless suggest that the oft-overlooked final transition may have an adaptive role in the Kv activation pathway. Paradoxically, a strictly thermal (insensitive to membrane voltage and to tension) entry to and exit from the conducting state would preserve a MS-Kv channel’s predominant role as V-sensitive conductance, allowing a Kv to be modulated by but not dominated by the mechanics of the bilayer. Since MS-Kv current in mechanosensory neurons was recently shown to be physiologically important [46] we also tested the two models in an excitability setting.

### Identity of the critical MS transition in Kv channels

Prokaryotic MS channels are acknowledged mechanical specialists [16,34] but Kv channels, too, have been called exquisite mechanosensors [19]. While we think this needs to be tempered, modulation of VGCs by membrane stress, reported first for calcium channels [67], occurs among members of all VGC families [4]. MS modulation of VGCs does not require that channels be co-expressed with auxiliary subunits (e.g., [61,68]) and it seems safe to expect that the deformable lipid/protein interface (and not, say, a cytoskeletal linker) will prove to be the “gating spring” [4,12,62] in all cases, but beyond that, no universal mechanism for VGC mechanosensitivity has emerged. In prokaryote MS channels, dramatic differences in cross-sectional areas and transverse thickness in closed versus open conformations underlie mechanosensitivity. In Kv channels, dimensional changes are far more minimal by comparison [69,70,71,72]. In-plane shape changes for comformations along the activation path (e.g. see Fig. 9 in Ref. [73]) would, however, require structural repacking at the adjacent bilayer interface. Given that stretch-induced left-shifts and right-shifts respectively are seen in the *g(V)* of Kv-WT and Kv-ILT a universal “∆ area” explanation for all MS-VCG already seems ruled out, though the possibility that a MS thermal transition could serve as such a mechanism was mooted [19]. A hypothetical universal mechanism would, further, need to explain how Nav and Kv1 but not Cav and Kv3 channels (all of which exhibit reversible stretch-induced increases in steady state *P*_*open*_ *(V)*) respond to stretch with accelerated current onset. We focused narrowly here, on two postulated mechanisms of mechanosensitivity in Shaker type Kv channels. The MS opening transition model of Schmidt et al, we termed *“LMS”*. We fleshed it out as a Markov model with standard rate constants (except for L, which needed MS-dependent rates) to allow the models to operate in the time domain. *g(V*,*t)* comparisons were done against “*vMS”* models in which a voltage-dependent closed-closed transition(s) near the open state underlies Kv mechanosensitivity [39].

### Does the model really matter?

Tabarean and Morris [39] indicated that a V-dependent closed-closded expansion near the open state in the range 2 to 9 nm^2^ would account for their data. Schmidt et al [19], alluding to atomic structural differences of closed and open pores near the inner bilayer leaflet, suggested a 3 to 4 nm^2^ area expansion during channel opening, emphasizing that to stabilize the open state, their model requires elevated membrane tension as much depolarization. The final arbiter of “openness” in their model (a *LMS*-Kv in our terms) is tension in the plane of the membrane, not transmembrane voltage. By contrast, for *vMS*-Kv models, tension modulates the rate of approach to the open state and shifts the *V*_*0*.*5*_ for activation without affecting the maximal *P*_*open*_.

In animal cells, plasma membranes, when not stressed by applied forces or osmotic swelling, are generally near-flaccid (neurons sustain small non-zero membrane tensions [32,74] and so the consequences of a MS opening as envisaged by Schmidt et al would be biologically non-trivial (e.g., see Fig. 8iib). The biophysical consequences would also be striking: Kv channel gating currents measured under rest tension conditions (near-flaccid) would yield true *Q*_*max*_ values but for true *gK*_*max*_ (several-fold beyond the apparent value measured at rest tension; see Fig. 5A) the V-clamped membrane would need to be subjected to untenably high stress. Kv channel density calculated using unitary conductance and rest tension *gK*_*max*_ would systematically be underestimated several-fold. In molecular models of V-sensing, attributions of gating charge per Kv channel would consequently be too high [75]. Inappropriate channel density measurements would also distort cell biological expectations regarding rates of Kv biogenesis and demands on trafficking machinery. Pharmacological quantities whose calculation assumes accurate channel density numbers (e.g. Kv/toxin binding interactions) would be assessed wrongly.

### The concerted V-dependent pre-opening transition

In the Kv tetramer, a concerted *V*-dependent pre-opening motion generates a centripetally acting force to untwist the intracellular bundle crossing, [76],[77],[66] yielding an “activated-closed” state (*C*_*6*_ in *Scheme 3*) [63]. Channel opening occurs at the next transition *C*_*6*_ *→O*. Comparison of the responses of Kv WT and Kv ILT channels to stretch can help discriminate between *vMS* or *LMS* mechanisms. In *vMS*, an area increase is assumed to occur at the *C*_*5*_ *→C*_*6*_ transition, while in *LMS* it occurs at *C*_*6*_ *→O*. In WT, stretch yields a *g(V)* left-shift, in ILT, a right shift [45]. Mechanical stretch, whether acting on the *C*_*5*_ *→C*_*6*_ or the *C*_*6*_ *→O* transition would decrease the enthalpy of the corresponding transition, and hence would yield a left shifted *g(V)*, the result observed for WT. ILT activation however exhibits *g(V)* right-shift during stretch [45]. This could be explained if stretch-deformation of the ILT/lipid interface increased the number of ways of being in *C*_*5*_ (= increased entropy and stability in *C*_*5*_) enough so that the free energy change at *C*_*5*_ *→C*_*6*_ is dominated not by the enthalpic decrease in *C*_*5*_ *→C*_*6*_ (as in WT) but by the entropic change. In summary, a net positive free energy change of this sort would result in a *g(V)* right-shift.

**Fig 9:**
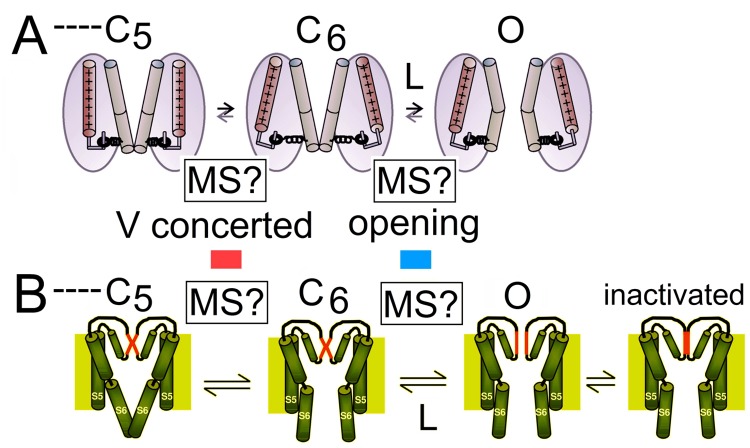
Two postulated versions for MS transitions and for the thermal open/closed transition in Kv channels. Cartoons **A** and **B** were originally designed not to address Kv mechanosensitivity, but to illustrate ideas about the concerted pre-opening and opening transitions. We have relabeled them showing, in each, how *vMS* puts the MS (expand-compact) transition at *C*_*5*_ *↔C*_*6*_, and likewise, in both, how *LMS* would put it at *C*_*6*_ *↔O*. In **A** (modified from Ref. [59]), *C*_*6*_ *↔O* is the opening of a bundle crossing gate while in **B** (modified from Ref. [79], release of the bundle crossing gate is necessary but not sufficient to allow for an *I*_*K*_ flow, which requires the opening of a selectivity filter gate (slow inactivation changes at the selectivity filter are also cartooned in **B**).

Co-operative bundle crossing movements close to Kv pore opening involve substeps [60,71,78]. A *V*-dependent in-plane expansion-substep (e.g., Fig. 9A) could readily underlie *vMS* but for *LMS* a late *V*-dependent concerted closed-closed transition would need to store elastic energy [4] without laterally expanding the channel (unlike cartoon Fig. 9A). In *LMS* expansion opens the permeation path. For *LMS*, a conundrum is that Kv closing’s demonstrable insensitivity to tension would imply a transition state essentially identical in area to the open state (Fig. 1Fv), thus implying *I*_*K*_ *(V)* onsets far more strongly accelerated by tension than observed. The identity of the final opening transition is, in any case, unsettled. A standard picture invokes bundle crossing motions (Fig. 9A) but another speculation (Fig. 9B; relabeled cartoon) [79] is that the closed bundle crossing allosterically constrains the selectivity filter (SF) in a non-conducting state while *V*-dependent opening of the bundle crossing relieves that constraint. Opening of the SF would be a final *V*-independent transition in the activation path. Voltage and tension working together to destabilize the penultimate closed state in a *vMS* version of Fig. 9B would be essentially as envisaged by Yifrach and Mackinnon [4].

### Operational sensitivity to membrane tension and to *V*_*m*_ in *LMS* versus *vMS* models

Terminating a *vMS*-Kv activation path with a strictly thermal closed/open transition diminishes the impact of bilayer mechanics on ion currents. A *LMS*-Kv is essentially a V-modulated MS channel whereas a *vMS*-Kv is a tension-modulated V-gated channel.

In a Hodgkin-Huxley *gK*, *P*_*open*_ approaches unity at large depolarizations, while, as per Hoshi et al [48], in our *vMS Schemes (1*,*2* or *3)*, maximal *P*_*open*_ = 0.8. In *LMS*, extreme membrane tensions allow *P*_*open*_ to approach unity. This could add scope for signalling about bilayer mechanics where extreme tension is relevant (e.g., in walled cells that move vertically in water columns and/or that experience osmotic turgor pressure changes). Although the data argue against *LMS-*Kv for Shaker type channels, data for Kv3 channels are not consistent with a *vMS* model; Kv3s show one key feature of a *LMS*-Kv (reversible MS increase in apparent *g*_*max*_) but have neither a MS-*tau* or a MS-*g(V)* shift [44]. Since smooth muscle maxi-Kv channels are strongly modulated by a dietary lipid [80] that appears to stabilize the open state it would be interesting to know if earlier reports of mechanosensitivity in similar channels [81] reflect MS modulation of a lipid-sensitive thermal C-O transition.

We concur with Schmidt et al that a channel such as they propose (the *LMS*-Kv model) would be “exquisitely mechanosensitive”, but for reasons noted above, the descriptor is apt only from certain perspectives. From a per-channel perspective, a *LMS* mechanism “disenfranchises” a portion of expressed channels. For example, in Fig. 8Ai (*L* = 1 ⇾ *L* = 1.76) the apparent half-*g*_*max*_ at rest tension is not 0.5 but 0.25, and even with near-lytic stretch, *LMS* precludes ~35% of channels from contributing current. Nevertheless, from a cellular excitability perspective, *LMS-*Kv channels would indeed be powerful mechano-effectors (Figs. 8Ai and 5Bii).

Our excitability modeling indicates, in fact, that a *LMS*-Kv could menace normal excitability (Fig. 8Biib). With *LMS*, excitability is extinguished by a 1.5 mV *g(V)* left-shift of only 30% of Kv channels, so if 100% of Kv channels were *LMS*-type, the most minor mechanical perturbation would likely be intolerable. However, cells have diverse MS channels [22] some of which are excitatory, and our basic excitability model ignored them. What our admittedly rudimentary excitability modeling demonstrates is that “stretch-induced” left-shifts of a MS component of *gK* will modulate evoked AP trains. Assuming an excitable membrane initially at rest tension (no pre-stress), *vMS* can produce graded MS modulation while *LMS* simply extinguishes excitability (as in Fig. 5Biib). Physiologically, all else being equal, a *LMS-*Kv would be a dramatically powerful MS brake on excitability, whereas a *vMS-*Kv would be a nuanced MS brake, conveying rather than supressing information about the strength and duration of mechanical deformations of the bilayer.

## Conclusion

The recent proposal that Kv channel mechanosensitivity resides with MS thermal (i.e. voltage-independent) closed-open transition has not, in our view, been established. Issues addressed here regarding recovery from slow inactivation, reversibility of MS responses, and tail current kinetics undermine the *LMS* case, as do the model’s implications for experimental determinations of Kv channel density and thence gating charge per channel. While we do not propose *vMS* as a universal mechanism for VGC mechanosensitivity (or even for all Kv channels), it remains a satisfying model for the MS responses of Shaker type Kv channels.

## Appendix: The two *gK(V*,*t)* subpopulations, one being an *MS-Kv*, for an HH excitability system

This Appendix explains how we subdivide the Kv conductance *gK(V*,*t)* in a Hodgkin Huxley (HH) excitability system, as discussed in Section **Excitability and a MS-Kv: *vMS* versus *LMS***. This allows us to implement different versions of an MS-Kv. The total K^+^ current density (in μA/cm^2^) in the two-population system is given by:

$$I_{K}=gK\left(f_{HH}n^{4}+f_{M}\frac{P_{o}}{P_{o,\max}(L_{0})}\right)(V-E_{K}),$$ [5]

where the potential terms *V* are in mV, and *f*_*HH*_ and *f*_*M*_ are respectively the fractions of the total conductance arising from HH-like and Markov like Kv channels at rest tension. The *f*_*HH*_ fraction obeys HH like kinetics and the *f*_*M*_ fraction *Scheme 1* like Markov kinetics (with *f*_*HH*_ + *f*_*M*_ = 1). In our calculations *f*_*M*_ was 0.3. *P*_*o*_ is the open probability of the *Scheme 1* channels; *P*_*o*,*max*_ *(L_0_)* is the maximum *P*_*o*_ at *L*_*0*_, the “rest tension” value of *L = k*_*o*_ */k*_*c*_. The total steady-state Kv conductance is given by:

$$g\left(V\right)=gK\left(f_{HH}n{\left(V\right)}^{4}+f_{M}\frac{P_{o}\left(V\right)}{P_{o,max}\left(L_{0}\right)}\right)$$ [6]

Its maximum value at *L = L*_*0*_, *gK* is the apparent *gK*_,*max*_. Only the *f*_*M*_ component is mechanosensitive. Increase in *L* leads to an increase in the maximal current density *g*_*K*_ (*V*—*E*_*K*_) of:

$$\Delta I_{K,\max}=gKf_{M}\frac{L-L_{0}}{L_{0}(1+L)}(V-E_{K}),$$ [7]

or an increase in the maximum conductance from *gK*, to *gK* + Δ*g*_*max*_ where:

$$\Delta g_{\max}=gKf_{M}\frac{L-L_{0}}{L_{0}(1+L)}.$$ [8]

The other physical constants for the Nav and Kv channels and the leak conductance are the same as in Table 1 of Ref. [65] (e.g. *gN*a = 120.0 mS/cm^2^, *g*K = 36.0 mS/cm^2^, *g*_leak_ = 0.25 mS/cm^2^, etc…, with equation constants adjusted to give time units as s). The kinetic parameters of the models (HH like and Markov) are given in Table 3.

## References

[1] Krepkiy D, Gawrisch K, Swartz KJ. Structural interactions between lipids, water and S1–S4 voltage-sensing domains. J Mol Biol. 2012;423:632–647. 10.1016/j.jmb.2012.07.015 22858867 PMC3616881

[2] Xu Y, Ramu Y, Shin HG, Yamakaze J, Lu Z. Energetic role of the paddle motif in voltage gating of Shaker K(+) channels. Nat Struct Mol Biol. 2013;20:574–581. 10.1038/nsmb.2535 23542156 PMC3777420

[3] Long SB, Campbell EB, Mackinnon R. Voltage sensor of Kv1.2: structural basis of electromechanical coupling. Science. 2005;309:903–908. 16002579 10.1126/science.1116270

[4] Morris CE. Voltage-gated channel mechanosensitivity: fact or friction? Front Physiol. 2011;2:25 10.3389/fphys.2011.00025 21660289 PMC3107450

[5] Morris CE. Pacemaker, potassium, calcium, sodium: stretch modulation of the voltage-gated channels In: Kohl P, Sachs F and Franz M, Cardiac eds. Mechano-Electric Coupling and Arrhythmias: from Pipette to Patient, 2nd Edn. Elsevier Saunders; 2011 pp43–49.

[6] Andersen OS, Koeppe 2nd RE. Bilayer thickness and membrane protein function: an energetic perspective. Annu. Rev. Biophys. Biomol. Struct. 2007;36:107–130. 17263662 10.1146/annurev.biophys.36.040306.132643

[7] Finol-Urdaneta RK, McArthur JR, Juranka PF, French RJ, Morris CE. Modulation of KvAP unitary conductance and gating by 1-alkanols and other surface active agents. Biophys J. 2010;98:762–772. 10.1016/j.bpj.2009.10.053 20197029 PMC2830433

[8] Morris CE, Boucher PA, Joos B. Left-shifted Nav channels in injured bilayer: primary targets for neuroprotective Nav antagonists? Front Pharmacol. 2012;3:19 10.3389/fphar.2012.00019 22375118 PMC3284691

[9] Gullingsrud J, Schulten K. Lipid bilayer pressure profiles and mechanosensitive channel gating. Biophys J. 2004;86:3496–3509. 15189849 10.1529/biophysj.103.034322 PMC1304254

[10] Zhuang X, Makover JR, Im W, Klauda JB. A systematic molecular dynamics simulation study of temperature dependent bilayer structural properties. Biochim Biophys. Acta. 2014;1838:2520–2529. 10.1016/j.bbamem.2014.06.010 24953542

[11] Soubias O, Teague WE Jr, Hines KG, Mitchell DC, Gawrisch K. Contribution of membrane elastic energy to rhodopsin function. Biophys J. 2010;99:817–824. 10.1016/j.bpj.2010.04.068 20682259 PMC2913204

[12] Phillips R, Ursell T, Wiggins P, Sens P. Emerging roles for lipids in shaping membrane-protein function. Nature. 2009;459:379–385. 10.1038/nature08147 19458714 PMC3169427

[13] Milescu M, Bosmans F, Lee S, Alabi AA, Kim JI, Swartz KJ. Interactions between lipids and voltage sensor paddles detected with tarantula toxins. Nat Struct Mol Biol. 2009;16:1080–1085. 10.1038/nsmb.1679 19783984 PMC2782670

[14] Schmidt D, Mackinnon R. Voltage-dependent K+ channel gating and voltage sensor toxin sensitivity depend on the mechanical state of the lipid membrane. Proc Natl Acad Sci USA. 2008;105:19276–19281. 10.1073/pnas.0810187105 19050073 PMC2614752

[15] Börjesson SI, Elinder F. An electrostatic potassium channel opener targeting the final voltage sensor transition. J Gen Physiol. 2011;137:563–577. 10.1085/jgp.201110599 21624947 PMC3105513

[16] Sukharev S1, Sachs F. Molecular force transduction by ion channels: diversity and unifying principles. J Cell Sci. 2012;125:3075–3083. 10.1242/jcs.092353 22797911 PMC3434843

[17] Gil Z1, Silberberg SD, Magleby KL. Voltage-induced membrane displacement in patch pipettes activates mechanosensitive channels. Proc Natl Acad Sci U S A. 1999;96:14594–14599. 10588750 10.1073/pnas.96.25.14594 PMC24481

[18] Morris CE, Juranka PF, Lin W, Morris TJ, Laitko U. Studying the mechanosensitivity of voltage-gated channels using oocyte patches. Methods Mol Biol. 2006;322:315–329. 16739733 10.1007/978-1-59745-000-3_22

[19] Schmidt D, del Mármol J, MacKinnon R. Mechanistic basis for low threshold mechanosensitivity in voltage-dependent K+ channel. Proc Natl Acad Sci U S A. 2012;109:10352–10357. 10.1073/pnas.1204700109 22675122 PMC3387069

[20] Opsahl LR, Webb WW. Lipid-glass adhesion in giga-sealed patch-clamped membranes. Biophys J. 1994;66:75–79. 8130347 10.1016/S0006-3495(94)80752-0 PMC1275665

[21] Morris CE, Sigurdson WJ. Stretch-inactivated ion channels coexist with stretch-activated ion channels. Science. 1989;243:807–809. 2536958 10.1126/science.2536958

[22] Morris CE. Why are so many ion channels mechanosensitive In: Sperelakis N.(Ed.), Cell Physiology Source Book, fourth ed Elsevier; 2011 pp. 493–505.

[23] Morris CE, Horn R. Failure to elicit neuronal macroscopic mechanosensitive currents anticipated by single-channel studies. Science 1991;251:1246–1249. 1706535 10.1126/science.1706535

[24] Small DL, Morris CE. Delayed activation of single mechanosensitive channels in *Lymnaea* neurons. Am J Physiol. 1994;267:C598–C606. 7521132 10.1152/ajpcell.1994.267.2.C598

[25] Tabarean I, Juranka PF, Morris CE. Membrane stretch affects gating modes of a skeletal muscle sodium channel. Biophys J. 1999;77:758–774. 10423424 10.1016/S0006-3495(99)76930-4 PMC1300370

[26] Wan X, Juranka P, Morris CE. Activation of mechanosensitive currents in traumatized membrane. Am J Physiol. 1999;276:C318–C327. 9950759 10.1152/ajpcell.1999.276.2.C318

[27] Hamill OP, McBride DW Jr. Induced membrane hypo/hyper-mechanosensitivity: a limitation of patch-clamp recording. Annu Rev Physiol. 1997;59:621–31. 9074780 10.1146/annurev.physiol.59.1.621

[28] Suchyna TM, Markin VS, Sachs F. Biophysics and structure of the patch and the gigaseal. Biophys J. 2009;97:738–747. 10.1016/j.bpj.2009.05.018 19651032 PMC2718145

[29] Morris CE, Juranka PF, Joos B. Perturbed voltage-gated channel activity in perturbed bilayers: Implications for ectopic arrhythmias arising from damaged membrane. Prog Biophys Mol Biol. 2012;110:245–256. 10.1016/j.pbiomolbio.2012.07.003 22846437

[30] Baumgart T, Das S, Webb WW, Jenkins JT. Membrane elasticity in giant vesicles with fluid phase coexistence. Biophys J. 2005;89:1067–1080. 15894634 10.1529/biophysj.104.049692 PMC1366592

[31] Sezgin E, Schwille P. Model membrane platforms to study protein-membrane interactions. Mol Membr Biol. 2012;29:144–54. 10.3109/09687688.2012.700490 22831167

[32] Van Essen DC. A tension-based theory of morphogenesis and compact wiring in the central nervous system. Nature. 1997;385:313–8. 9002514 10.1038/385313a0

[33] Otway R, Vandenberg JI, Guo G, Varghese A, Castro ML, Liu J, et al Stretch-sensitive KCNQ1 mutation. A link between genetic and environmental factors in the pathogenesis of atrial fibrillation? J Am Coll Cardiol. 2007;49:578–86. 17276182 10.1016/j.jacc.2006.09.044

[34] Shaikh S, Cox CD, Nomura T, Martinac B. Energetics of gating MscS by membrane tension in azolectin liposomes and giant spheroplasts. Channels (Austin). 2014;8:321–326. 24758942 10.4161/chan.28366 PMC4203733

[35] Ledwell JL, Aldrich RW. Mutations in the S4 region isolate the final voltage-dependent cooperative step in potassium channel activation. J Gen Physiol. 1999;113:389–414. 10051516 10.1085/jgp.113.3.389 PMC2222902

[36] Smith-Maxwell CJ, Ledwell JL, Aldrich RW. Role of the S4 in cooperativity of voltage-dependent potassium channel activation. J Gen. Physiol. 1998;111:399–420. 9482708 10.1085/jgp.111.3.399 PMC2217113

[37] Zagotta WN, Hoshi T, Aldrich RW. Shaker potassium channel gating. III: Evaluation of kinetic models for activation. J Gen Physiol. 1994;103:321–362. 8189208 10.1085/jgp.103.2.321 PMC2216839

[38] Boucher PA, Morris CE, Joós B. Mechanosensitive closed-closed transitions in large membrane proteins: osmoprotection and tension damping. Biophys J. 2009;97:2761–2770. 10.1016/j.bpj.2009.08.054 19917230 PMC2776245

[39] Tabarean IV, Morris CE. Membrane stretch accelerates activation and slow inactivation in Shaker channels with S3–S4 linker deletions. Biophys J. 2002;82:2982–2994. 12023221 10.1016/S0006-3495(02)75639-7 PMC1302086

[40] Sukharev SI, Markin VS. Kinetic model of the bacterial large conductance mechanosensitive channel. Biologie`eskie Membrany. 2001;18:440–445.

[41] Lecar H, Morris CE. Biophysics of mechanotransduction In: Rubanyi GM ed. Mechanoreception by the Vascular Wall. Mount Kisco, NY: Futura Publications; 1993 pp 1–11.

[42] Markin VS, Sachs F. Thermodynamics of mechanosensitivity. Phys Biol. 2004;1:110–124. 16204828 10.1088/1478-3967/1/2/007

[43] Gu CX, Juranka PF, Morris CE. Stretch-activation and stretch-inactivation of Shaker-IR, a voltage-gated K+ channel. Biophys J. 2001;80:2678–2693. 11371444 10.1016/S0006-3495(01)76237-6 PMC1301455

[44] Laitko U, Morris CE. Membrane tension accelerates rate-limiting voltage-dependent activation and slow inactivation steps in a Shaker channel. J Gen Physiol. 2004;123:135–154. 14744987 10.1085/jgp.200308965 PMC2217428

[45] Laitko U, Juranka PF, Morris CE. Membrane stretch slows the concerted step prior to opening in a Kv channel. J Gen Physiol. 2006;127:687–701. 16735754 10.1085/jgp.200509394 PMC2151533

[46] Hao J, Padilla F, Dandonneau M, Lavebratt C, Lesage F, Noël J, et al Kv1. 1 channels act as mechanical brake in the senses of touch and pain. Neuron. 2013;77:899–914. 10.1016/j.neuron.2012.12.035 23473320

[47] Otway R, Vandenberg JI, Guo G, Varghese A, Castro ML, Liu J, et al Stretch-sensitive KCNQ1 mutation. A link between genetic and environmental factors in the pathogenesis of atrial fibrillation? J Am Coll Cardiol. 2007;49:578–586. 17276182 10.1016/j.jacc.2006.09.044

[48] Hoshi T, Zagotta WN, Aldrich RW. Shaker potassium channel gating. I: Transitions near the open state. J Gen Physiol. 1994;103:249–278. 8189206 10.1085/jgp.103.2.249 PMC2216835

[49] Gonzalez C, Rosenman E, Bezanilla F, Alvarez O, Latorre R. Modulation of the Shaker K+ channel gating kinetics by the S3–S4 linker. J Gen Physiol. 2000;115:193–207. 10653896 10.1085/jgp.115.2.193 PMC2217197

[50] Schoppa NE, McCormack K, Tanouye MA, Sigworth FJ. The size of gating charge in wild-type and mutant Shaker potassium channels. Science. 1992;255:1712–1715. 1553560 10.1126/science.1553560

[51] Schoppa NE, Sigworth FJ. Activation of shaker potassium channels. I. Characterization of voltage-dependent transitions. J Gen Physiol. 1998;111:271–294. 9450944 10.1085/jgp.111.2.271 PMC2222764

[52] Noceti F, Baldelli P, Wei X, Qin N, Toro L, Birnbaumer L, et al Effective gating charges per channel in voltage dependent K^+^ and Ca^2+^ channels. J Gen Physiol. 1996;108:143–155. 8882860 10.1085/jgp.108.3.143 PMC2229320

[53] Seoh SA, Sigg D, Papazian DM, Bezanilla F. Voltage sensing residues in the S2 and S4 segments of the Shaker K+ channel. Neuron. 1996;16:1159–1167. 8663992 10.1016/s0896-6273(00)80142-7

[54] Sigg D, Stefani E, Bezanilla F. Gating current noise produced by elementary transitions in Shaker potassium channels. Science. 1994;264:578–582. 8160016 10.1126/science.8160016

[55] Loots E, Isacoff EY. Molecular coupling of S4 to a K^+^ channel’s slow inactivation gate. J Gen Physiol. 2000;116:623–636. 11055991 10.1085/jgp.116.5.623 PMC2229480

[56] Conti F, Fioravanti R, Segal JR, Stuhmer W. Pressure dependence of the potassium currents of squid giant axon. J Membr Biol. 1982;69:35–40. 7120362 10.1007/BF01871239

[57] Lin W, Laitko U, Juranka PF, Morris CE. Dual stretch responses of mHCN2 pacemaker channels: accelerated activation, accelerated deactivation. Biophys J. 2007;92:1559–1572. 17142286 10.1529/biophysj.106.092478 PMC1796836

[58] Männikkö R, Pandey S, Larsson HP, Elinder F. Hysteresis in the voltage dependence of HCN channels: conversion between two modes affects pacemaker properties. J Gen Physiol. 2005;125:305–326. 15710913 10.1085/jgp.200409130 PMC2234019

[59] Pathak M, Kurtz L, Tombola F, Isacoff, E. The cooperative voltage sensor motion that gates a potassium channel. J Gen Physiol. 2005;125:57–69. 15623895 10.1085/jgp.200409197 PMC1414780

[60] Gagnon DG, Bezanilla F. The contribution of individual subunits to the coupling of the voltage sensor to pore opening in Shaker K channels: effect of ILT mutations in heterotetramers. J Gen Physiol. 2010;136:555–568. 10.1085/jgp.201010487 20974773 PMC2964516

[61] Banderali U, Juranka PF, Clark RB, Giles WR, Morris CE. Impaired stretch modulation in potentially lethal cardiac sodium channel mutants. Channels (Austin). 2010;4:12–21. 20090423 10.4161/chan.4.1.10260

[62] Morris CE, Juranka PF. Nav channel mechanosensitivity: activation and inactivation accelerate reversibly with stretch. Biophys J. 2007;93:822–833. 17496023 10.1529/biophysj.106.101246 PMC1913161

[63] del Camino D, Kanevsky M, Yellen G. Status of the intracellular gate in the activated-not-open state of shaker K+ channels. J Gen Physiol. 2005;12:419–428. 10.1085/jgp.200509385 PMC1794167 16260836

[64] Hodgkin AL, Huxley AF. A quantitative description of membrane current and its application to conduction and excitation in nerve. J Physiol. 1952;117:500–544. 12991237 10.1113/jphysiol.1952.sp004764 PMC1392413

[65] Boucher PA, Joos B, Morris CE. Coupled left-shift of Nav channels: modeling the Na^+^-loading and dysfunctional excitability of damaged axons. J Comput Neuro. 2012;33:301–319. 10.1007/s10827-012-0387-7 22476614

[66] Yifrach O, MacKinnon R. Energetics of pore opening in a voltage-gated K^+^ channel. Cell. 2002;111:231–239. 12408867 10.1016/s0092-8674(02)01013-9

[67] Langton PD. Calcium channel currents recorded from isolated myocytes of rat basilar artery are stretch sensitive. J Physiol. 1993;471:1–11. 8120799 10.1113/jphysiol.1993.sp019887 PMC1143948

[68] Calabrese B, Tabarean IV, Juranka P, Morris CE. Mechanosensitivity of N-type calcium channel currents. Biophys J. 2002;83:2560–2574. 12414690 10.1016/S0006-3495(02)75267-3 PMC1302342

[69] Bezanilla F. How membrane proteins sense voltage. Nat Rev Mol Cell Biol. 2008;9:323–332. 10.1038/nrm2376 18354422

[70] Horn R. Uncooperative voltage sensors. J Gen Physiol. 2009;133:463–466. 10.1085/jgp.200910236 19398774 PMC2712971

[71] Faure E, Thompson C, Blunck R. Do lipids show state-dependent affinity to the voltage-gated potassium channel KvAP? J Biol Chem. 2014;289:16452–16461 10.1074/jbc.M113.537134 24742679 PMC4047412

[72] Li Q, Wanderling S, Sompornpisut P, Perozo E. Structural basis of lipid-driven conformational transitions in the KvAP voltage-sensing domain. Nat Struct Mol Biol. 2014;21:160–166. 10.1038/nsmb.2747 24413055 PMC3946318

[73] Lacroix JJ, Hyde HC, Campos FV, Bezanilla F. Moving gating charges through the gating pore in a Kv channel voltage sensor. Proc Natl Acad Sci U S A. 2014;111:E1950–1959. 10.1073/pnas.1406161111 24782544 PMC4024920

[74] Batchelder EL, Hollopeter G, Campillo C, Mezanges X, Jorgensen EM, Nassoy P, et al Membrane tension regulates motility by controlling lamellipodium organization. Proc Natl Acad Sci U S A. 2011;108:11429–11434. 10.1073/pnas.1010481108 21709265 PMC3136290

[75] Alvarez O, Gonzalez C, Latorre R. Counting channels: a tutorial guide on ion channel fluctuation analysis. Adv Physiol Educ. 2002;26:327–41. 12444005 10.1152/advan.00006.2002

[76] Mannuzzu LM, Isacoff EY. Independence and cooperativity in rearrangements of a potassium channel voltage sensor revealed by single subunit fluorescence. J Gen Physiol. 2000;115:257–268. 10694254 10.1085/jgp.115.3.257 PMC2217208

[77] Zheng J, Vankataramanan L, Sigworth FJ. Hidden Markov model analysis of intermediate gating steps associated with the pore gate of shaker potassium channels. J Gen Physiol. 2001;118:547–564. 11696611 10.1085/jgp.118.5.547 PMC2233839

[78] Kalstrup T, Blunck R. Dynamics of internal pore opening in Kv channels probed by a fluorescent unnatural amino acid. Proc Natl Acad Sci U S A. 2013;110:8272–8277. 10.1073/pnas.1220398110 23630265 PMC3657800

[79] Labro AJ, Snyders DJ. Being flexible: the voltage-controllable activation gate of Kv channels. Frontiers Pharmacol. 2012;3:168 22993508 10.3389/fphar.2012.00168 PMC3440756

[80] Hoshi T, Tian Y, Xu R, Heinemann SH, Hou S. Mechanism of the modulation of BK potassium channel complexes with different auxiliary subunit compositions by the omega-3 fatty acid DHA. Proc Natl Acad Sci U S A. 2013;110:4822–4827. 10.1073/pnas.1222003110 23487786 PMC3607020

[81] Dopico AM, Kirber MT, Singer JJ, Walsh JV Jr. Membrane stretch directly activates large conductance Ca(2+)-activated K+ channels in mesenteric artery smooth muscle cells. Am J Hypertens. 1994;7:82–9. 8136116 10.1093/ajh/7.1.82

